# Supplementary material for: R-loop proximity proteomics identifies a role of DDX41 in transcription-associated genomic instability
Source: Nat Commun. 2021 Dec 16;12:7314. doi: 10.1038/s41467-021-27530-y (PMC8677849; doi:10.1038/s41467-021-27530-y)
Supplement: Supplementary file 1 — Supplementary Information [file 41467_2021_27530_MOESM1_ESM.pdf]

Supplementary information

R-loop proximity proteomics identifies a role of DDX41 in transcription-associated genomic instability

Thorsten Mosler[1], Francesca Conte[1], Gabriel M. C. Longo[1], Ivan Mikicic[1], Nastasja Kreim[1], Martin M. Möckel[1], Giuseppe Petrosino[1], Johanna Flach[2], Joan Barau[1], Brian Luke[1,3], Vassilis Roukos[1], Petra Beli[1,3,*]

[1]Institute of Molecular Biology (IMB), Mainz, Germany

[2]Department of Hematology and Oncology, Medical Faculty Mannheim of the Heidelberg University, Mannheim, Germany

[3]Institute of Developmental Biology and Neurobiology (IDN), Johannes Gutenberg-Universität, Mainz, Germany

[*]Correspondence should be addressed to p.beli@imb-mainz.de

This PDF file contains:

Supplementary figure 1: Mapping R-loop-proximal proteome on native chromatin

Supplementary figure 2: DDX41 depletion leads to replication stress and genomic instability

Supplementary figure 3: DDX41 opposes R-loop accumulation

Supplementary figure 4: Genome-wide analysis of DDX41 binding to chromatin and R-loops in DDX41 knockdown U2OS cells

Supplementary figure 5: Genome-wide analysis of DSBs and R-loops in DDX41 knockdown HCT116 cell

Lists of antibodies, siRNAs, oligonucleotides used in this study

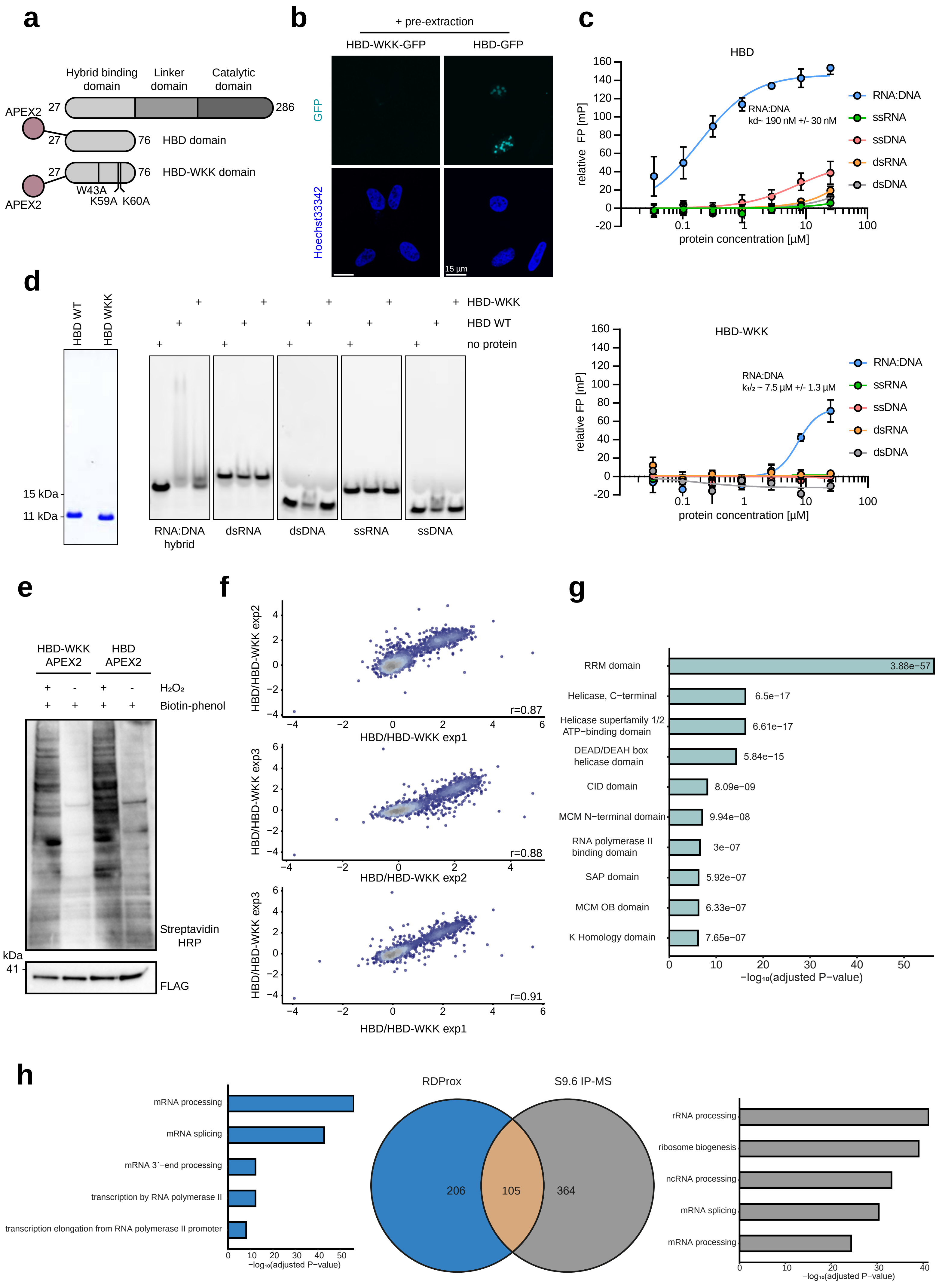

a
Hybrid binding domain
Linker domain
Catalytic domain
APEX2
27
286
76
HBD domain
HBD-WKK domain
W43A
K59A K60A
b
+ pre-extraction
HBD-WKK-GFP
HBD-GFP
GFP
Hoechst33342
15 µm
c
HBD
relative FP [mP]
RNA:DNA
kd~ 190 nM +/- 30 nM
RNA:DNA
ssRNA
ssDNA
dsRNA
dsDNA
protein concentration [µM]
HBD-WKK
RNA:DNA
k1/2 ~ 7.5 µM +/- 1.3 µM
d
HBD WT
HBD WKK
HBD-WKK
HBD WT
no protein
15 kDa
11 kDa
RNA:DNA hybrid
dsRNA
dsDNA
ssRNA
ssDNA
e
HBD-WKK APEX2
HBD APEX2
H2O2
Biotin-phenol
kDa
41
Streptavidin HRP
FLAG
f
HBD/HBD-WKK exp1
HBD/HBD-WKK exp2
HBD/HBD-WKK exp3
r=0.87
r=0.88
r=0.91
g
RRM domain 3.88e−57
Helicase, C−terminal 6.5e−17
Helicase superfamily 1/2 ATP−binding domain 6.61e−17
DEAD/DEAH box helicase domain 5.84e−15
CID domain 8.09e−09
MCM N−terminal domain 9.94e−08
RNA polymerase II binding domain 3e−07
SAP domain 5.92e−07
MCM OB domain 6.33e−07
K Homology domain 7.65e−07
−log10(adjusted P−value)
h
mRNA processing
mRNA splicing
mRNA 3´−end processing
transcription by RNA polymerase II
transcription elongation from RNA polymerase II promoter
−log10(adjusted P−value)
RDProx
S9.6 IP-MS
206
105
364
rRNA processing
ribosome biogenesis
ncRNA processing
mRNA splicing
mRNA processing
−log10(adjusted P−value)

Supplementary figure 1: Mapping R-loop-proximal proteome on native chromatin

a. Schematic representation of M27-RNaseH1 domains. The wild type version of hybrid binding domain (HBD) of RNaseH1 was cloned as well as the mutant with indicated WKK point mutations to abrogate the binding function. HBD and HBD-WKK were fused to the ascorbate peroxidase APEX2 on the N-terminus. Numbers indicate amino acid positions.
b. Immunofluorescence analysis of the retention of GFP-tagged HBD or HBD-WKK on chromatin after pre-extraction with 0.4% NP-40 for 30 min on ice. DNA was counterstained with Hoechst33342 (blue), GFP signal (cyan). Representative images of n = 2 biologically independent experiments. Scale bar represents 15 µm.
c. Fluorescence polarization (FP) assay of wild type HBD and HBD-WKK mutant. Titrated domains incubated together with indicated 20 nM 6-FAM-conjugated oligonucleotide substrates. Data of n = 3 independent experiments with individually thawed proteins are represented as mean +/- standard deviation. Colored lines represent either a Michalis-Menten or a sigmoidal fit. Resulting kD or $k_{1/2}$ values for the association to the RNA-DNA hybrid substrate are displayed. Source data are provided as a Source Data file.
d. Electro Mobility Shift Assay (EMSA) using 25 µM purified HBD or HBW-WKK mutant and indicated 6-FAM-conjugated oligonucleotides. Protein inputs shown left. Data derived from n = 1 experiment. Source data are provided as a Source Data file.
e. Representative western blot of biotinylated proteins in U2OS whole cell extracts after HBD-APEX2 or HBD-WKK-APEX2 proximity labeling from n = 2 biologically individual experiments. Source data are provided as a Source Data file.
f. Multi-scatter plots indicating the correlation between the n = 3 biologically independent RDProx SILAC experiments in HEK293T cells. Pearson correlation is indicated below each plot. Coloring indicates the density of the population (blue=less dense, grey= high density).
g. PFAM InterPro Domain enrichment analysis using EnrichR. Adjusted p-values were derived using two-sided Fisher´s exact test with Bonferroni correction.
h. GO Biological Process analysis of protein groups identified in S9.6 immunoprecipitation-based mass spectrometry screen (Cristini et al., 2018) and RDProx Tier 1. The 5 GO-BP terms with the lowest adjusted p-value (two-sided Fisher´s exact test, Bonferroni correction) are shown.

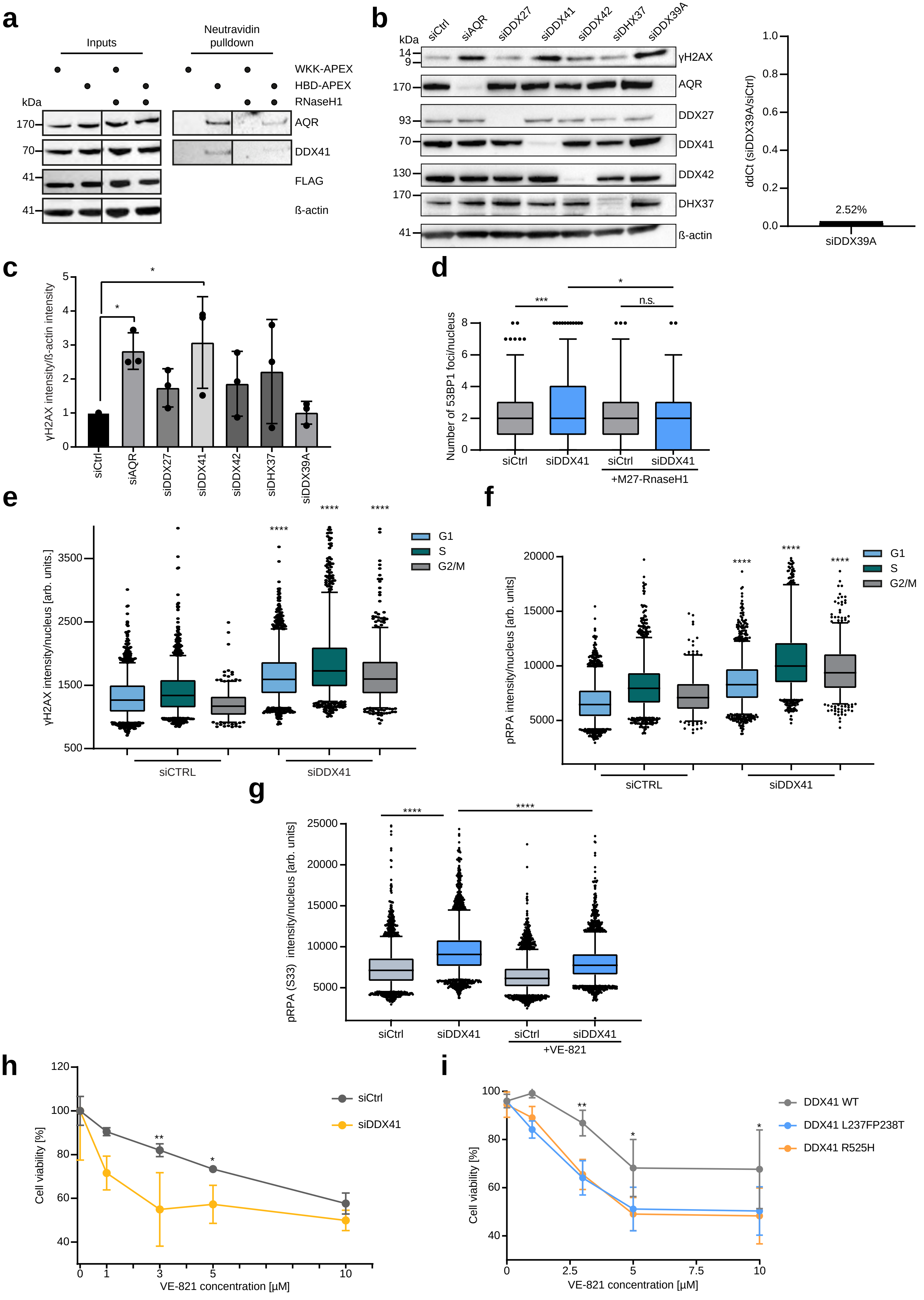
a
Inputs
Neutravidin pulldown
WKK-APEX
HBD-APEX
RNaseH1
kDa
170
70
41
41
AQR
DDX41
FLAG
ß-actin
b
kDa
siCtrl
siAQR
siDDX27
siDDX41
siDDX42
siDHX37
siDDX39A
14
9
170
93
70
130
170
41
γH2AX
AQR
DDX27
DDX41
DDX42
DHX37
ß-actin
ddCt (siDDX39A/siCtrl)
1.0
0.8
0.6
0.4
0.2
0.0
2.52%
siDDX39A
c
γH2AX intensity/ß-actin intensity
5
4
3
2
1
0
*
*
siCtrl
siAQR
siDDX27
siDDX41
siDDX42
siDHX37
siDDX39A
d
Number of 53BP1 foci/nucleus
8
6
4
2
0
***
*
n.s.
siCtrl
siDDX41
siCtrl
siDDX41
+M27-RnaseH1
e
γH2AX intensity/nucleus [arb. units.]
3500
2500
1500
500
****
****
****
G1
S
G2/M
siCTRL
siDDX41
f
pRPA intensity/nucleus [arb. units]
20000
15000
10000
5000
****
****
****
G1
S
G2/M
siCTRL
siDDX41
g
pRPA (S33) intensity/nucleus [arb. units]
25000
20000
15000
10000
5000
****
****
siCtrl
siDDX41
siCtrl
siDDX41
+VE-821
h
Cell viability [%]
120
100
80
60
40
**
*
0
1
3
5
10
VE-821 concentration [µM]
siCtrl
siDDX41
i
Cell viability [%]
100
80
60
40
**
*
*
0
2.5
5
7.5
10
VE-821 concentration [µM]
DDX41 WT
DDX41 L237FP238T
DDX41 R525H

Supplementary figure 2: DDX41 depletion leads to replication stress and genomic instability

a. RDProx-Western blot analysis AQR and DDX41 in U2OS cells +/- expression of M27-RNaseH1. Data from n = 1 experiment. Source data are provided as a Source Data file.
b. Representative Western blot of yH2AX in U2OS cells after indicated knockdown. Bar plot shows qPCR after DDX39A knockdown. Mean value of the ddCt values between siDDX39A and a control knockdown against GAPDH control of n = 1 biologically individual experiment is plotted. Data are represented as the mean +/- standard deviation of 2 technical replicates. Source data are provided as a Source Data file.
c. Quantification of the ratio between γH2AX intensity and ß-actin. Data of n = 3 biologically independent experiments is represented as the mean +/- standard deviation. P-values (p = 0.0297, p = 0.2627, p = 0.0177, p = 0.2627, p = 0.1876, p = 0.8433) derived using Kruskal-Wallis test with Benjamini-Hochberg correction for multiple comparisons. Source data are provided as a Source Data file.
d. Immunofluorescence analysis of 53BP1 foci 48 h after indicated knockdowns in U2OS cells +/- expression of M27-RNaseH1-GFP. Boxplot limits indicate the 25th to 75th percentile, whiskers the 5th -95th percentile and the center line the median. Dots represent outliers. Three outlier data points above 8 foci/cell are cut off. Representative data of n = 2 biologically independent experiments. P-values (p = 0.008, p = 0.0455, p = 0.3005) derived from n > 170 cells using one-way-ANOVA with Tukey correction for multiple comparisons. Source data are provided as a Source Data file.
e. Immunofluorescence analysis of γH2AX after 48 h knockdown with the indicated siRNAs in different cell cycle stages: G1 (blue), S (green), G2/M (grey). Centerline of box plots represents the median, whiskers indicate the 10th-90th percentile and box limits the 25th-75th percentile. Dots represent outliers. Representative data of n = 2 biologically independent experiments. P-values (p < 0.0001, p < 0.0001, p < 0.0001) derived by one-way-ANOVA with Tukey correction for multiple comparisons. Source data are provided as a Source Data file.
f. Same as in S2e, but cells were stained for pRPA (Ser33). P-values (p < 0.0001, p < 0.0001, p < 0.0001) derived by one-way-ANOVA with Tukey correction for multiple comparisons. Source data are provided as a Source Data file.
g. Immunofluorescence analysis of pRPA (Ser33) in U2OS cells after indicated knockdowns for 48 h and 6 h treatment with DMSO or 10 µM VE-821. Boxplot limits indicate the 25th to 75th percentile, whiskers the 5th -95th percentile and the center line the median. Outliers are represented by dots. P-values (p < 0.0001, p < 0.0001) derived by one-way-ANOVA with Tukey correction for multiple comparisons. N = 2. Source data are provided as a Source Data file.
h. Cell titer blue assay in U2OS cells after indicated knockdowns. Data from n = 3 biologically independent experiments is represented as mean +/- standard deviation. P-values (p = 0.033, p = 0.0038) derived using two-way ANOVA with Tukey correction for multiple comparisons. Source data are provided as a Source Data file.
i. Cell titer blue assay in OCI-AML3 cells after expression of DDX41 WT, R525H or L237FP238T mutants. Data from n = 3 biologically independent experiments is represented as mean +/- standard deviation. P-values (p = 0.0044, p = 0.0078, p = 0.0374, p = 0.0179, p = 0.338, p = 0.0162) derived using two-way ANOVA with Tukey correction for multiple comparisons. Source data are provided as a Source Data file.

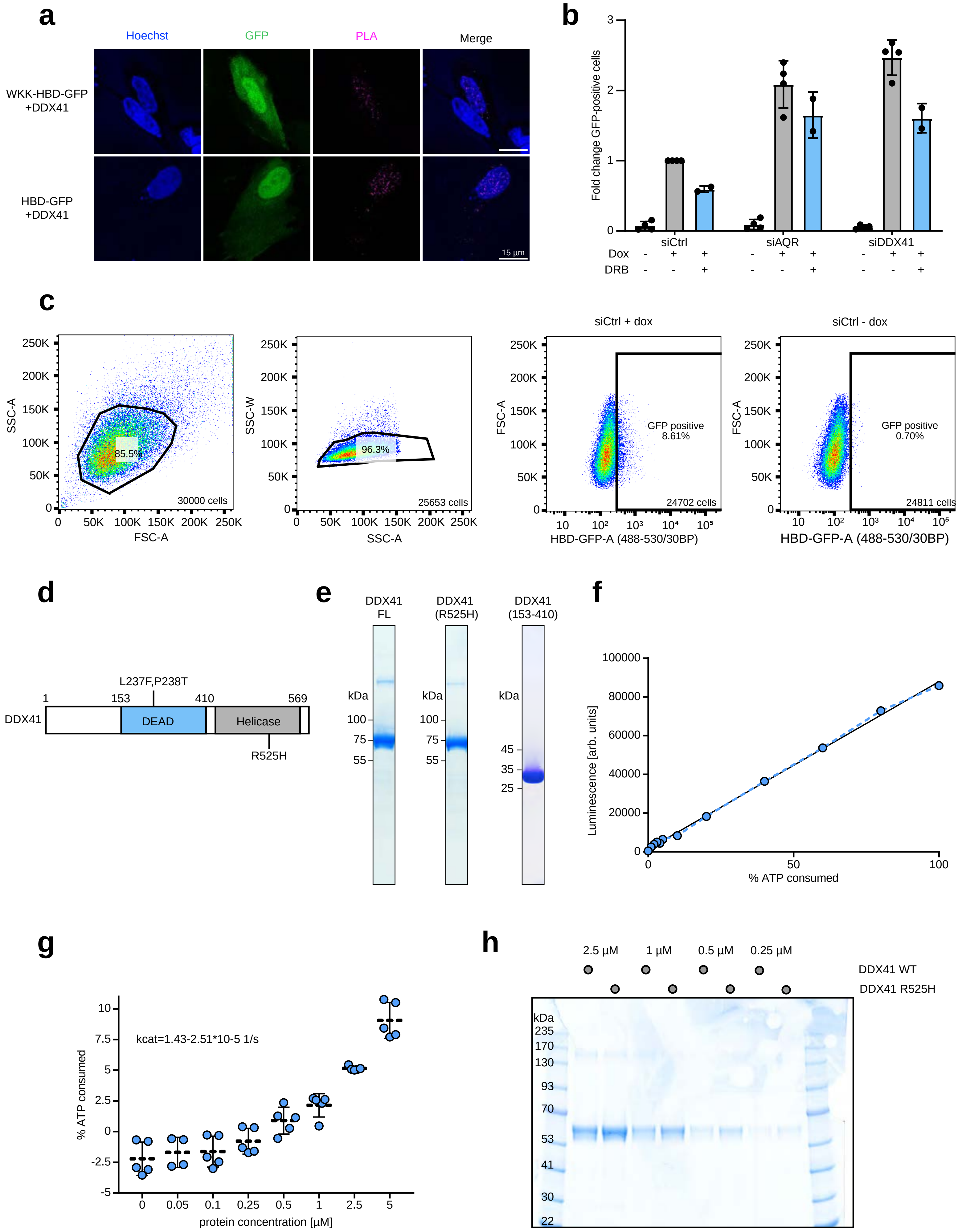

a
Hoechst
GFP
PLA
Merge
WKK-HBD-GFP +DDX41
HBD-GFP +DDX41
15 µm
b
Fold change GFP-positive cells
siCtrl
siAQR
siDDX41
Dox
DRB
c
siCtrl + dox
siCtrl - dox
SSC-A
FSC-A
SSC-W
SSC-A
85.5%
30000 cells
96.3%
25653 cells
GFP positive 8.61%
24702 cells
GFP positive 0.70%
24811 cells
HBD-GFP-A (488-530/30BP)
d
DDX41
L237F,P238T
DEAD
Helicase
R525H
1
153
410
569
e
DDX41 FL
DDX41 (R525H)
DDX41 (153-410)
kDa
f
Luminescence [arb. units]
% ATP consumed
g
kcat=1.43-2.51*10-5 1/s
% ATP consumed
protein concentration [µM]
h
2.5 µM
1 µM
0.5 µM
0.25 µM
DDX41 WT
DDX41 R525H
kDa

Supplementary figure 3: DDX41 opposes R-loop accumulation

a. Representative images of Proximity Ligation assay (PLA) between endogenous DDX41 and exogenously expressed HBD-GFP or HBD-WKK-GFP. Data derived from n = 1 individual experiment. Scale bar represents 15 µm.
b. Bar plot displays the quantification of the HBD retention assay in U2OS cells analyzed by flow cytometry 48 after indicated knockdowns +/- 2 h treatment with DMSO or 10 µM DRB. Data are represented as mean +/- standard deviation. Dots represent individual measurements. Experiments were performed: n = 4 for knockdowns, n = 2 for DRB controls. Source data are provided as a Source Data file.
c. Representative flow cytometry gating strategy used in Supplementary figure 3b. Data was derived from n = 4 biologically independent experiments.
d. Schematic representation of full length DDX41 domain organization. Pathogenic AML/MDS variants are indicated. Numbers represent corresponding amino acid positions.
e. Coomassie-stained gel of 6his-tagged DDX41 full length, R525H and 153-410 mutant after expression and purification from SF9 insect cells.
f. Standard curve for the interpolation of values obtained from the ADP-Glo assay. ATP and ADP were mixed in various ratios and further processed together with ADP-Glo samples. Activity of the luciferase was increased with increased ADP concentration in a linear manner. Source data are provided as a Source Data file.
g. ADP-Glo assay measuring ATP hydrolysis of full length DDX41 in n = 5 independent experiments using individually thawed proteins. Titrated DDX41 was incubated together with 100 nM RNA-DNA hybrid substrate with an ssDNA overhang and 5 µM ATP for 60 minutes at 37°C. ATP to ADP conversion was measured by luciferase activity. Results are displayed as percentage of consumed ATP compared to total ATP, based on an interpolation against a standard curve. Dots indicate individual measurements. Data are represented as mean +/- standard deviation. Source data are provided as a Source Data file.
h. Coomassie-stained gel of 6his-tagged DDX41 full length and R525H mutant protein inputs used for the RNA-DNA hybrid unwinding assay in Figure 3f. Representative data of n = 3 experiments with individually thawed proteins.

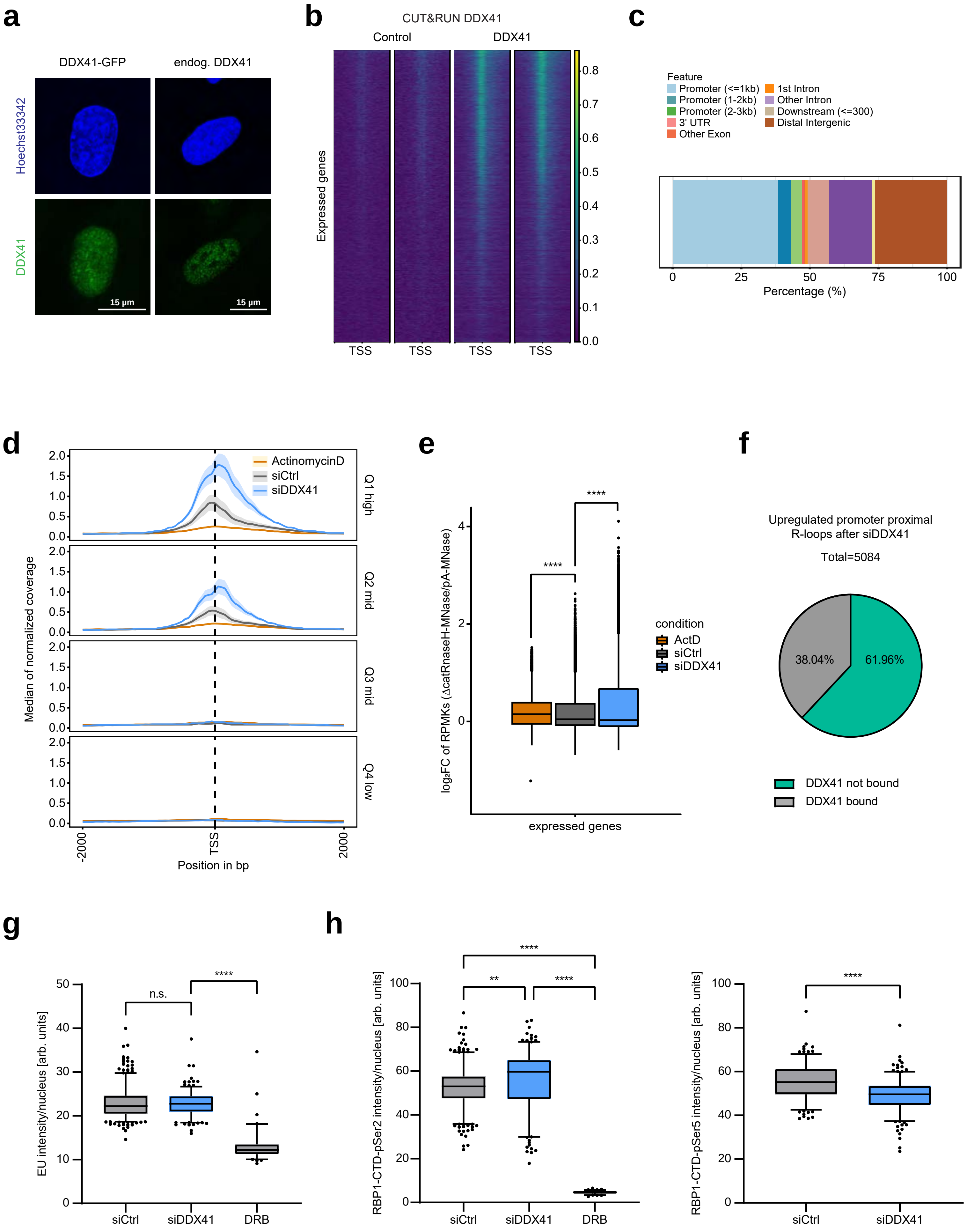
a
DDX41-GFP
endog. DDX41
Hoechst33342
DDX41
15 µm
15 µm
b
CUT&RUN DDX41
Control
DDX41
Expressed genes
TSS
TSS
TSS
TSS
0.8
0.7
0.6
0.5
0.4
0.3
0.2
0.1
0.0
c
Feature
Promoter (<=1kb)
Promoter (1-2kb)
Promoter (2-3kb)
3' UTR
Other Exon
1st Intron
Other Intron
Downstream (<=300)
Distal Intergenic
0
25
50
75
100
Percentage (%)
d
ActinomycinD
siCtrl
siDDX41
Q1 high
Q2 mid
Q3 mid
Q4 low
Median of normalized coverage
-2000
TSS
2000
Position in bp
e
log₂FC of RPMKs (ΔcatRnaseH-MNase/pA-MNase)
condition
ActD
siCtrl
siDDX41
****
****
expressed genes
f
Upregulated promoter proximal
R-loops after siDDX41
Total=5084
38.04%
61.96%
DDX41 not bound
DDX41 bound
g
EU intensity/nucleus [arb. units]
n.s.
****
siCtrl
siDDX41
DRB
h
RBP1-CTD-pSer2 intensity/nucleus [arb. units]
****
**
****
siCtrl
siDDX41
DRB
RBP1-CTD-pSer5 intensity/nucleus [arb. units]
****
siCtrl
siDDX41

Supplementary figure 4: Genome-wide analysis of DDX41 binding to chromatin and R-loops in DDX41 knockdown U2OS cells

a. Representative immunofluorescence images from n = 1 experiment of the subcellular localization of doxycycline-inducible GFP-tagged DDX41 and endogenous (endog.) DDX41. Scale bars represent 15 µm.
b. Heat maps of CUT&RUN signal from -2kilo bases (kb)/+2kb around the transcription start site (TSS) of expressed genes sorted by expression level.
c. Bar plot displaying the feature distribution of all peaks called by MACS2 in the MapR experiment in n = 3 biologically independent experiments from U2OS cells after 48 h knockdown with control siRNA. Consensus peaks were constructed using the intersection of peaks in the replicates per group (Control, DDX41 knockdown) and the union of the resulting peaks were used as a consensus peak set. Proportion of the individual feature is represented in the indicated colors.
d. MapR in U2OS cells performed in n = 3 biologically independent experiments after 48 h knockdown with control siRNA (grey), DDX41 siRNA (blue) or treatment with 4 µM Actinomycin D for 6 hours (orange). Metagene profiles of MapR signal ranging from -2kilo bases (kb)/+2kb around the transcription start site (TSS) of all expressed genes based on RNA-sequencing analysis of U2OS cells, divided in 4 quantiles. Shadows indicate the standard error of the median between replicates.
e. The $\log_2$ fold change (FC) of the reads per kilo base per million mapped reads (RPMK) of MapR signal between the ΔcatRNaseH-MNase and the untargeted pA-MNase obtained +/-1kb around the transcription start site (TSS) of all expressed genes based on RNA-sequencing. Mean RPMKs between n = 3 biologically independent experiments are shown. Whiskers represent the 5th-95th percentile, limits the 25th-75th percentile, the center line the median and individual dots depict outliers. P-values ($p < 0.0001$, $p < 0.0001$) were derived using two-sided paired Welch´s t-test.
f. Pie chart shows the proportion of R-loop gains in promoter regions that were also found to associate with GFP-DDX41 in CUT&RUN experiments in U2OS cells.
g. Confocal microscopy analysis of EU intensity in U2OS cells after transfection for 48 h with control or DDX41 siRNA or DRB treatment for 3 h. Center line within the box represents the median, whiskers the 5th-95th percentile, limits the 25th-75th percentile and dots indicate outliers. At least 200 cells per condition were measured across n = 1 experiment. P-values ($p = 0.761$, $p < 0.0001$) derived using one-Way-ANOVA with Tukey correction for multiple comparisons. Source data are provided as a Source Data file.
h. Confocal microscopy analysis of RBP1-CTD pSer2 or pSer5 intensity in U2OS cells after transfection for 48 h with control or DDX41 siRNA or DRB treatment for 3 h. Center line within the box represents the median, whiskers the 5th-95th percentile, limits the 25th-75th percentile and dots indicate outliers. At least 200 cells per condition were measured across n = 1 experiment. P-values ($p = 0.0095$, $p < 0.0001$, $p < 0.0001$) were calculated using one-way-ANOVA with Tukey correction for multiple comparisons. Ser5: p-value < 0.0001 derived using two-sided student´s t-test. Source data are provided as a Source Data file.

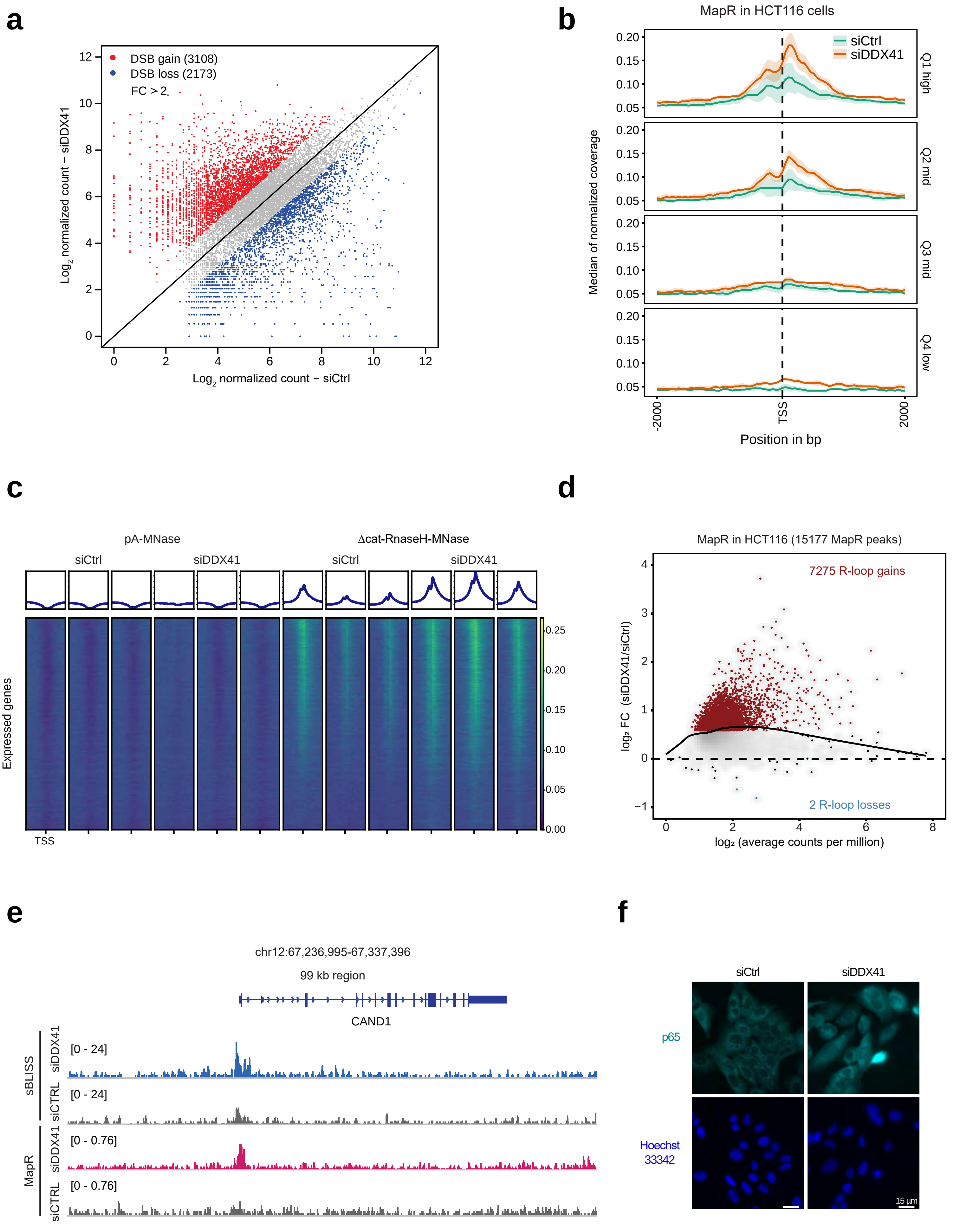
a
DSB gain (3108)
DSB loss (2173)
FC > 2
Log2 normalized count – siDDX41
Log2 normalized count – siCtrl
b
MapR in HCT116 cells
siCtrl
siDDX41
Q1 high
Q2 mid
Q3 mid
Q4 low
Median of normalized coverage
-2000
TSS
2000
Position in bp
c
pA-MNase
Δcat-RnaseH-MNase
siCtrl
siDDX41
siCtrl
siDDX41
Expressed genes
TSS
d
MapR in HCT116 (15177 MapR peaks)
7275 R-loop gains
2 R-loop losses
log2 FC (siDDX41/siCtrl)
log2 (average counts per million)
e
chr12:67,236,995-67,337,396
99 kb region
CAND1
sBLISS
siDDX41
[0 - 24]
siCTRL
[0 - 24]
MapR
siDDX41
[0 - 0.76]
siCTRL
[0 - 0.76]
f
siCtrl
siDDX41
p65
Hoechst 33342
15 µm

Supplementary figure 5: Genome-wide analysis of DSBs and R-loops in DDX41 knockdown HCT116 cells

a. Scatter plot of log_2 normalized counts of the peaks detected in control and DDX41 knockdown cells measured by sBLISS in n = 2 biologically independent experiments. Fold change (FC) > 2 was taken to determine genomic regions with DSB gains and DSB loss.
b. MapR performed in n = 3 biological replicates in HCT116 cells after 48 h knockdown with control siRNA (green) or DDX41 siRNA (red). Metagene profiles of MapR signal ranging from -2kb/+2kb around the transcription start site (TSS) of all expressed genes based on the published GRO-sequencing analysis in HCT116 cells divided in 4 quantiles. Shadows indicate the standard error of the median between replicates.
c. Heat maps showing the distribution of the MapR signal of all expressed genes for pA-MNase and ΔcatRNaseH-MNase conditions after 48 h control or DDX41 knockdown. MapR signal was sorted based on gene expression level obtained from the previously published GRO-sequencing analysis from high to low. The region -2kb/+ 2kb around the TSS is individually displayed for each biological replicate. Metagene profiles are outlined above the associated heat maps.
d. Scatter plot of MapR regions in HCT116 cells. Consensus regions were constructed using the intersection of peaks for the replicates in each condition (siCtrl and siDDX41). The union of these regions was used for further analysis and quantification of the coverage/fold change (FC). The mean log_2 FC between siCtrl and siDDX41 is plotted against the log_2 average counts per million representing the coverage. Genomic regions that are differentially regulated (FC>2) are highlighted in red (up) or in blue (down).
e. Representative snapshot of a genomic region showing accumulation of R-loops and DSBs profiled by MapR and sBLISS, respectively, in HCT116 cells.
f. Confocal microscopy of p65 after 48 h control knockdown or DDX41 knockdown in U2OS cells. Representative images of p65 staining (cyan) and DNA counterstaining with DAPI are displayed on the left. Quantification is shown in Figure 5h. Data derived from n = 2 biologically independent experiments. Scale bars represent 15 µm.

List of antibodies used in this study

Protein name	Product number/clone	Origin	Dilution (WB/IF)
γH2AX	A300-081A-M	Bethyl	(1:1000/1:500)
53BP1	MAB3802/ BP13	Millipore	(-/1:200)
pRPA (S33)	A300-246A-M	Bethyl	(-/1:200)
DDX41	15076/ D3F1Z	Cell Signaling	1:1000
GFP	sc-9996/ B-2	Santa Cruz	1:1000
S9.6	ENH001/ S9.6	Kerafast	1:10000
dsDNA	ab27156/ 35I9 DNA	Abcam	1:1000
AQR	A302-547A	Bethyl	1:2000
DDX42	SAB1407136	Sigma	1:1000
DDX39A	SAB2700315	Sigma	1:1000
FLAG M2	F1804/ M2	Sigma	1:2000
B-Actin	A2228/ AC-74	Sigma	1:10000
DHX37	A300-856A-M	Bethyl	1:1000
BrdU (mouse)	347580/ B44	BD Bioscience	1:100
BrdU(rat)	ab6326/BU1/75(ICR1)	Abcam	1:500
Anti-mouse Cy3.5	Ab6946	Abcam	1:100
Anti-rat Cy5	Ab6565	Abcam	1:100
P65	sc-372/ C-20	Santa Cruz	(-/1:200)
Phospho-Rpb1-CTD (Ser2)	13499/ E1Z3G	Cell signaling	(-/1:200)
Phospho-Rpb1-CTD (Ser5)	13523/ D9N5I	Cell signaling	(-/1:200)

List of siRNA used in this study

Gene name	Sequence 5'-3' or origin
AQR	CUGAAUAUGGCGGUGUAGU
DDX41	L-010394-00-0005 – Horizon Discovery
DDX42	L-012393-01-0005 – Horizon Discovery
DHX37	L-019073-00-0005– Horizon Discovery
DDX39A	L-004920-01-0005 – Horizon Discovery
DDX27	L-013635-01-0005 – Horizon Discovery
Si control pool	D-001820-10 – Horizon Discovery

List of oligos used in this study

Construct	Sequence 5'-3'
DDX41-fw-qPCR	GTCCGTGAAAGAGCAGATGGAG
DDX41-rev-qPCR	GTAGCGACAGATGTCTAGGCTG
HBD-backbone-fw	cctcctcacggcatagaacatggtggagcctgcttttttgtacaaagttgg
HBD-backbone-rev	cctttgtcaggaaatctgcaagcgacccagctttcttgtacaaagt
HBD-insert-fw	ccaactttgtacaaaaaagcaggctccaccatgttctatgccgtgaggagg
HBD-insert rev	actttgtacaagaaagctgggtcgcttgcagatttcctgacaaagg
HBD-W43A-fw	ctttctgaccgcgaatgagtgcagagcacaggtggaccg
HBD-W43A-rev	accccggtcttgcggccc
HBD-K59A-K60A-fw	tgccagatttgcggcgtttgccacagaggatgaggc
HBD-K59A-K60A-rev	gcaggaaaccggtccacc
DDX41-R525H-fw	CGCACCGGGCACTCGGGAAAC
DDX41-R525H_rev	GCCAATCCGGTGTACATAGTTCTC
DDX41-L237F-P238T-fw	TGTTCACGTTTACCGTCATCATG
DDX41-L237FP238T-rev	CCAGTGTCTTGCCTGAAC
DDX41-3C-rev	ACCGGGCCCCTGGAACAGAAC
DDX41-L153-fw	TTCTGTTCCAGGGGCCCGGTCTGAGCATGTCTGAAGAGC
DDX41-Q410-rev	gtgctcgagtgcggccgctcactggatgacatccaggctg
DNA-12mer-fw	GACACCTGATTC-6-FAM
DNA-12mer-rev	GAATCAGGTGTC
RNA-12mer-fw	GACACCTGATTC-6-FAM
RNA-12mer-rev	GAATCAGGTGTC
DNA-38mer-IBFQ	TAAAACAAAACAAAACAAAACAAAATCTTTACGGTGCT-IBFQ
RNA-13mer-6-FAM	6-FAM-AGCACCGUAAAGA
DNA-38mer	TAAAACAAAACAAAACAAAACAAAATCTTTACGGTGCT
