# Supplementary material for: R-loop proximity proteomics identifies a role of DDX41 in transcription-associated genomic instability
Source: Nat Commun. 2021 Dec 16;12:7314. doi: 10.1038/s41467-021-27530-y (PMC8677849; doi:10.1038/s41467-021-27530-y)
Supplement: Supplementary file 2 — Description of Additional Supplementary Files [file 41467_2021_27530_MOESM2_ESM.pdf]

Description of Additional Supplementary Files

File Name: Supplementary Data 1
Description: All protein groups identified in Tier1 and Tier2 by RDProx in HEK293T cells with indicated mean log2 fold changes and FDR.

File Name: Supplementary Data 2
Description: Annotated CUT&RUN peaks indicative of GFP-DDX41 binding in U2OS cells. Only peaks present in both biological replicate experiments are included in the table.

File Name: Supplementary Data 3
Description: Annotated MapR peaks with a FC > 2 (R-loop gain and loss) in U2OS cells. Distal intergenic peaks are not included in the table.

File Name: Supplementary Data 4
Description: Gene list from RNA-sequencing analysis in U2OS cells with indicated log2 fold change and FDR derived using a Wald test and Benjamini-Hochberg correction for multiple testing.

File Name: Supplementary Data 5
Description: Annotated sBLISS peaks used for the fold change analysis in HCT116 cells

File Name: Supplementary Data 6
Description: Annotated MapR peaks with a FC > 1.5 (R-loop gain and loss) in HCT116 cells. Distal intergenic peaks are not included in the table.
