# Supplementary material for: R-loop proximity proteomics identifies a role of DDX41 in transcription-associated genomic instability
Source: Nat Commun. 2021 Dec 16;12:7314. doi: 10.1038/s41467-021-27530-y (PMC8677849; doi:10.1038/s41467-021-27530-y)
Supplement: Supplementary file 10 — Reporting Summary [file 41467_2021_27530_MOESM10_ESM.pdf]

nature portfolio

Corresponding author(s): Petra Beli

Last updated by author(s): 19.11.2021

Reporting Summary

Nature Portfolio wishes to improve the reproducibility of the work that we publish. This form provides structure for consistency and transparency in reporting. For further information on Nature Portfolio policies, see our Editorial Policies and the Editorial Policy Checklist.

Statistics

For all statistical analyses, confirm that the following items are present in the figure legend, table legend, main text, or Methods section.

n/a	Confirmed	
☐	☒	The exact sample size (*n*) for each experimental group/condition, given as a discrete number and unit of measurement
☐	☒	A statement on whether measurements were taken from distinct samples or whether the same sample was measured repeatedly
☐	☒	The statistical test(s) used AND whether they are one- or two-sided<br>*Only common tests should be described solely by name; describe more complex techniques in the Methods section.*
☒	☐	A description of all covariates tested
☐	☒	A description of any assumptions or corrections, such as tests of normality and adjustment for multiple comparisons
☐	☒	A full description of the statistical parameters including central tendency (e.g. means) or other basic estimates (e.g. regression coefficient) AND variation (e.g. standard deviation) or associated estimates of uncertainty (e.g. confidence intervals)
☐	☒	For null hypothesis testing, the test statistic (e.g. *F*, *t*, *r*) with confidence intervals, effect sizes, degrees of freedom and *P* value noted<br>*Give P values as exact values whenever suitable.*
☒	☐	For Bayesian analysis, information on the choice of priors and Markov chain Monte Carlo settings
☒	☐	For hierarchical and complex designs, identification of the appropriate level for tests and full reporting of outcomes
☐	☒	Estimates of effect sizes (e.g. Cohen's *d*, Pearson's *r*), indicating how they were calculated

Our web collection on statistics for biologists contains articles on many of the points above.

Software and code

Policy information about availability of computer code

Data collection	No software was used for data collection.
Data analysis	RStudio (1.3.959); STRING database (v 11.0b); Cytoscape (v 3.2.1), REACTOME (v 2021); PANTHER (16.0); EnrichR (2016 update); CometScore (TriTek Corp., v 2.0); Fiji/ImageJ (v 1.51), MaxQuant (1.5.2.8); GraphPad Prism (Graphpad Software, Inc. 7.04), blc2fastq (v 2.19); bowtie2 (v 2.3.4); MACS2 (v 2.1.2); Diffbind (v 3.0.5); ClusterProfiler (v 3.18); DeepTools (v 3.4.1); ChIPSeeker (v 1.26); STAR (2.7); featureCounts (v 1.6); Bioconductor (v 2.46); DESeq2 (v 1.26); Harmony High-Content Imaging and Analysis Software (v 4.4, PerkinElmer); edgeR (v 3.32.1); BWA-MEM (v 0.7.15); bedtools (v 2.27.0); Cutadapt (v 1.18); FastQC (v 0.1.2); ChIPpeakanno (v 3.28.0); GenomicRanges (v 1.46.0); rtracklayer (v 1.54.0); GenomicFeatures (v 1.46.1)

For manuscripts utilizing custom algorithms or software that are central to the research but not yet described in published literature, software must be made available to editors and reviewers. We strongly encourage code deposition in a community repository (e.g. GitHub). See the Nature Portfolio guidelines for submitting code & software for further information.

Data

Policy information about availability of data

All manuscripts must include a data availability statement. This statement should provide the following information, where applicable:
- Accession codes, unique identifiers, or web links for publicly available datasets
- A description of any restrictions on data availability
- For clinical datasets or third party data, please ensure that the statement adheres to our policy

The fasta file containing the human proteome (released in 04/2018) used for analysis of raw MS data using MaxQuant was retrieved from UniprotKB: https://www.uniprot.org/proteomes/UP000005640. HCT116 GRO-seq data to determine gene expression levels for MapR analysis was retrieved from GEO (GSM2296622):

https://www-ncbi-nlm-nih-gov.ezproxy.u-pec.fr/geo/query/acc.cgi?acc=GSM2296622. RNA-Seq, MapR and BLISS data generated in this study have been deposited in the GEO database under accession code GSE168173 (https://www.ncbi.nlm.nih.gov/geo/query/acc.cgi?acc=GSE168173). The mass spectrometry-based proteomics data have been deposited to the ProteomeXchange Consortium via the PRIDE partner repository with the dataset identifier PXD024517 (https://www.ebi.ac.uk/pride/archive/projects/PXD024517). Source data are provided with this paper.

Field-specific reporting

Please select the one below that is the best fit for your research. If you are not sure, read the appropriate sections before making your selection.

- [x] Life sciences
- [ ] Behavioural & social sciences
- [ ] Ecological, evolutionary & environmental sciences

For a reference copy of the document with all sections, see nature.com/documents/nr-reporting-summary-flat.pdf

Life sciences study design

All studies must disclose on these points even when the disclosure is negative.

Sample size	No calculation or statistics were used to determine sample size. Sample size was determined based on the bast practices established for proteomics (SILAC-MS) and genomics (RNA-seq, MapR, CUT&RUN, sBLISS) experiments and were chosen such that statistical significance can be established. Quantitative proteomic SILAC-based experiments were performed in n = 3 biologically independent experiments. Genomic experiments (RNA-seq, MapR, CUT&RUN, sBLISS) were performed in at least 2 (n = 2 for sBLISS and CUT&RUN and n = 3 for RNA-seq and MapR) biologically independent experiments.
Data exclusions	No data was excluded from the analysis.
Replication	Biologically independent experiments were performed as stated in the corresponding figure legends. All attempts of replication were successful.
Randomization	Randomization was not feasible for this type of molecular biology, biochemistry, proteomics and genomics experiments. All samples were analyzed or treated in the same manner.
Blinding	Randomization was not feasible for this type of molecular biology, biochemistry, proteomics and genomics experiments. All samples were analyzed or treated in the same manner.

Reporting for specific materials, systems and methods

We require information from authors about some types of materials, experimental systems and methods used in many studies. Here, indicate whether each material, system or method listed is relevant to your study. If you are not sure if a list item applies to your research, read the appropriate section before selecting a response.

Materials & experimental systems

n/a	Involved in the study	
[ ]	[x]	Antibodies
[ ]	[x]	Eukaryotic cell lines
[x]	[ ]	Palaeontology and archaeology
[x]	[ ]	Animals and other organisms
[x]	[ ]	Human research participants
[x]	[ ]	Clinical data
[x]	[ ]	Dual use research of concern

Methods

n/a	Involved in the study	
[ ]	[x]	ChIP-seq
[ ]	[x]	Flow cytometry
[x]	[ ]	MRI-based neuroimaging

Antibodies

Antibodies used	All antibodies, their description and their clone name for monoclonal antibodies is provided as Table 1 of the manuscript.
Validation	Polyclonal rabbit yH2AX antibody quality control was performed by Bethyl: Western blot using an antibody concentration of 0.04 µg/ml based on whole cell extracts from 3T3 cells revealed specific band after 100 µm Etoposide treatment that was not present in the mock control. Increased nuclear intensity based on immunofluorescence microscopy of Neocarzinostatin-treated Hela cells compared to untreated. https://www.bethyl.com/product/A300-081A-M<br><br>Mouse monoclonal 53BP1 antibody validated by Millipore: specific foci formation after 10 Gy irradition of Hela cells compared to untreated control. https://www.merckmillipore.com/DE/de/product/Anti-53BP1-Antibody-clone-BP13,MM_NF-MAB3802<br><br>Rabbit polyclonal pRPA (S33) antibody validated by Bethyl: specific Western blot band from Hela whole cell lysates treated with 100 µM Etoposide for 16 h in comparison to untreated control. https://www.bethyl.com/product/A300-246A-M

Rabbit monoclonal DDX41 antibody validated by Cell Signaling: Overexpression of full length DDX41 in HEK293 cells resulted in increased specific band on Western blot. Immunoprecipitation enriched specific band. https://beli.imb.uni-mainz.de/uploads/collection_management/antibody/abPB224.pdf

Mouse monoclonal antibody was tested by SantaCruz: Direct near-infrared western blot analysis of GFP expression in human cells recombinant GFP fusion protein. Immunofluorescence staining of methanol-fixed COS cells transfected with GFP fusion protein showing cytoplasmic staining. https://beli.imb.uni-mainz.de/uploads/collection_management/antibody/abPB22.pdf

We validated mouse monoclonal S9.6 antibody specificity by dot blot. Specific signal was lost after pre-treatment of genomic DNA with E.coli Rnase H in vitro.

Mouse monoclonal dsDNA antibody was validated by Abcam: Measurements by immuno-CE yielded KD's of 0.71 µM and 0.09 µM, for the interaction of this antibody with ss- and dsDNA, respectively. https://www.abcam.com/ds-dna-antibody-35i9-dna-bsa-and-azide-free-ab27156.html

Rabbit polyclonal AQR antibody was validated by Bethyl: specific Western blot band after immunoprecipitation of AQR from Hela whole cell lysates compared to no band form IgG control. https://www.bethyl.com/product/A302-547A#

Mouse polyclonal DDX42 antibody was validated by Sigma-Aldrich: Specific Western blot band after overexpression of DDX42 in 293T cells. https://www.sigmaaldrich.com/DE/de/product/sigma/sab1407136

Rabbit polyclonal DDX39A antibody was validated by Sigma-Aldrich: Specific Wester blot band at correct height and nuclear IF signal for various human cell lines. https://www.sigmaaldrich.com/DE/de/product/sigma/sab2700315?images=true&context=product

We validated specificity for monoclonal mouse FLAG antibody by expressing FLAG-tagged HBD-APEX: specific band in case of overexpression in comparison to untransfected control.

Mouse monoclonal ß-actin antibody tested by Sigma-Aldrich: Immunofluorescence analysis in HS-68 cells showed actin filament staining. https://www.sigmaaldrich.com/DE/de/product/sigma/a5441?context=product

Rabbit polyclonal DHX37 antibody was validated by loss of Western blot band with specific DHX37 siRNA knockdown.

Mouse monoclonal BrdU antibody was validated by immunofluorescence analysis in U2OS cells +/- BrdU incorporation.

Rat monoclonal BrdU antibody was validated by abcam: Hela cells treated with 10 µm BrdU for 24 h showed specific signal in IF and flow cytometry with the antibody. https://www.abcam.com/brdu-antibody-bu175-icr1-bsa-and-azide-free-ab264079.html

Mouse monoclonal p65 antibody was validated by SantaCruz: specific band at correct size in Hela and MCF7 cells. https://www.scbt.compnfkappabp65antibodya-12gclid=Cj0KCQiAkNiMBhCxARIsAIDDKNVAz_NBhAAQ39m8hWBs7ZsDYn_XizMcZcjDlfBgXWW_AWnOSE7xL4aApBBEALw_wcB

Rabbit monoclonal Rbp1-CTD (Ser2) antibody was validated by Cell signaling: Immunoprecipitation of Rpb1 from HeLa cells resulted in specifc Western blot band that was absent in the IgG control. https://www.cellsignal.com/products/primary-antibodies/phospho-rpb1-ctd-ser2-e1z3g-rabbit-mab/13499

Rabbit monoclonal Rbp1-CTD (Ser5) antibody was validated by Cell signaling: Immunoprecipitation of Rpb1 from HeLa cells resulted in specifc Western blot band that was absent in the IgG control. https://www.cellsignal.com/products/primary-antibodies/phospho-rpb1-ctd-ser5-d9n5i-rabbit-mab/13523

Eukaryotic cell lines

Policy information about cell lines

Cell line source(s)	U2OS and HEK293T cells were directly obtained from ATCC (American Type Culture Collection). HCT116 and OCI-AMl3 cells were obtained from DSMZ (German collection of Microorganisms and Cell Cultures GmbH).
Authentication	U2OS cells were authenticated by ATCC based on karyotyping, antigen expression, expression markers, gene expression and STR profiling.<br>HEK293T were authenticated by ATCC based on STR profiling.<br>HCT116 were authenticated by DSMZ by karyotyping, STR profiling, species PCR and morphology.<br>OCI-AML3 were authenticated by DSMZ based on STR profiling, morphology, cytogenics, species PCR and antigen expression.
Mycoplasma contamination	All cell lines were tested routinely for Mycoplasma contamination using a PCR-based test and were Mycoplasma negative.
Commonly misidentified lines (See ICLAC register)	None of these cell lines are used in this study.

ChIP-seq

Data deposition

- [x] Confirm that both raw and final processed data have been deposited in a public database such as GEO.
- [x] Confirm that you have deposited or provided access to graph files (e.g. BED files) for the called peaks.

Data access links<br>*May remain private before publication.*	Genomics data are available on GEO under the reference GSE168173.
Files in database submission	The database submission (GSE183083, Superseries GSE158173) contains the compressed fastq files for read 1 and read 2 as well as the sequencing depth normalised coverage tracks (bigwig). The bigwig tracks do not use the reads mapped on the mitochondrion for normalisation of sequencing depth. The following samples were submitted:<br>imb_beli_2021_07_05_nanobodyGFP_control_crosslink_rep1, imb_beli_2021_07_06_nanobodyGFP_control_crosslink_rep2, imb_beli_2021_07_07_nanobodyGFP_DDX41_crosslink_rep1, imb_beli_2021_07_08_nanobodyGFP_DDX41_crosslink_rep2
Genome browser session<br>(e.g. UCSC)	http://genome-euro.ucsc.edu/s/%40imb%2Dmainz.de/U2OS_cut_and_run

Methodology

Replicates	Two biological replicates of uninduced control cells against doxycycline-induced DDX41-GFP expressing cells with high reproducibility.
Sequencing depth	13.7 mio - 14.6 mio reads per sample were obtained. 4.7 mio - 7 mio reads were uniquely mapped for each sample. Read 1: 34nt/ Read 2: 49nt/ Index i7: 8nt, paired-end.
Antibodies	GFP-nanobody-MNase was produced in-house and used for the CUT&RUN experiment. Purification of the nanobody is described in the methods section.
Peak calling parameters	Peakcalling was done using macs (v2.1.2) with the following parameter adjustments "-g hs --min-length 150 --format BAMPE".
Data quality	The raw sequencing reads were trimmed for non template nucleotides (15 bp on the read2) and adapter sequences were removed before mapping using Cutadapt (v.1.18). Adapter content before and after trimming was checked using FastQC (v. 0.11.8). After mapping mapping statistics and distribution of reads over the genome were taken into account. The data did show a high amount of reads mapped to the mitochondrion. Reads mapped to the miochondrion were excluded for the read depth normalisation of the bigwig files (produced by Deeptools v3.4.1) to avoid biases caused by the different amount of reads mapped to the mitochondrion.<br>Amount peaks for FDR 0.05 and FC 5: 12438 (replicate 1) and 8283 (replicate 2)
Software	The first 15 bases of the second read of the cut and run data were removed and the data was adapter trimmed using Cutdapt (v.1.18). The data was mapped against hg38 using bowtie2 (v2.3.4) and filtered for uniquely mapping reads. Peakcalling was done using MACS2 (v2.1.2) with the following parameters "-g hs --min-length 150 --format BAMPE --keep-dup auto". The data was further analysed using R (v. 4.03) and Bioconductor (v 3.11) packages within R scripts and the Deeptools (v3.4.1) suite. The most important packages Bioconductor packages used are ChIPSeeker, ChIPpeakanno, GenomicRanges, rtracklayer and GenomicFeatures.

Flow Cytometry

Plots

Confirm that:

- [x] The axis labels state the marker and fluorochrome used (e.g. CD4-FITC).
- [x] The axis scales are clearly visible. Include numbers along axes only for bottom left plot of group (a 'group' is an analysis of identical markers).
- [x] All plots are contour plots with outliers or pseudocolor plots.
- [x] A numerical value for number of cells or percentage (with statistics) is provided.

Methodology

Sample preparation	U2OS dox-inducible cell line expressing HBD-GFP, 1µg/ml doxycycline for 48h, control cells 3h 10 µM DRB, 0.05% Triton X-100 for 3 min pre-extraction, 4% PFA fixation
Instrument	BD LSRFortessa SORP
Software	Aquisition with FACS-DIVA 8.0.2, analysis with FlowJo 10.7
Cell population abundance	Starting population consisted of 30,000 cells. After gating, GFP-positive cells were determined from the relevant cell population consisting of roughly 25,000 cells.

Gating strategy

SSC-A/FSC-A, SSC-W/SSC-A, FSC-A/GFP

☒ Tick this box to confirm that a figure exemplifying the gating strategy is provided in the Supplementary Information.
